# Ethnic Inequalities in Mortality: The Case of Arab-Americans

**DOI:** 10.1371/journal.pone.0029185

**Published:** 2011-12-28

**Authors:** Abdulrahman M. El-Sayed, Melissa Tracy, Peter Scarborough, Sandro Galea

**Affiliations:** 1 Department of Epidemiology, Columbia University, New York, New York, United States of America; 2 Department of Public Health, University of Oxford, Oxford, United Kingdom; 3 College of Physicians and Surgeons, Columbia University, New York, New York, United States of America; 4 Center for Social Epidemiology and Population Health, Department of Epidemiology, University of Michigan School of Public Health, Ann Arbor, Michigan, United States of America; University of Massachusetts Medical School, United States of America

## Abstract

**Background:**

Although nearly 112 million residents of the United States belong to a non-white ethnic group, the literature about differences in health indicators across ethnic groups is limited almost exclusively to Hispanics. Features of the social experience of many ethnic groups including immigration, discrimination, and acculturation may plausibly influence mortality risk. We explored life expectancy and age-adjusted mortality risk of Arab-Americans (AAs), relative to non-Arab and non-Hispanic Whites in Michigan, the state with the largest per capita population of AAs in the US.

**Methodology/Principal Findings:**

Data were collected about all deaths to AAs and non-Arab and non-Hispanic Whites in Michigan between 1990 and 2007, and year 2000 census data were collected for population denominators. We calculated life expectancy, age-adjusted all-cause, cause-specific, and age-specific mortality rates stratified by ethnicity and gender among AAs and non-Arab and non-Hispanic Whites. Among AAs, life expectancies among men and women were 2.0 and 1.4 years lower than among non-Arab and non-Hispanic White men and women, respectively. AA men had higher mortality than non-Arab and non-Hispanic White men due to infectious diseases, chronic diseases, and homicide. AA women had higher mortality than non-Arab and non-Hispanic White women due to chronic diseases.

**Conclusions/Significance:**

Despite better education and higher income, AAs have higher age-adjusted mortality risk than non-Arab and non-Hispanic Whites, particularly due to chronic diseases. Features specific to AA culture may explain some of these findings.

## Introduction

Inequalities in health have been demonstrated in high-income contexts since the advent of the quantitative assessment of population health metrics. In the United States (US), however, health research concerned with health inequalities between continental population groups has been dominated by studies concerned with “racial” disparities in health. Races are differentiated biologically via phenotypic similarities (e.g., skin color) within races and differences across races [1]. Several authors have criticized the use of this metric in epidemiology [1]–[5]. Race does not imply shared ancestral ancestry, language, or culture and therefore groups vastly heterogeneous populations. For example, both a recent Egyptian immigrant to the US and an Anglo-Saxon whose family had immigrated to the US five generations prior would both be considered “White” according to the US Office of Management and the Budget, which defines the race category “White” as “persons originating in Europe, the Middle East and North Africa (OMB) [6]. “Ethnicity” as a population health metric implies differentiation on the basis of shared place of origin. While heterogeneity within groups may also challenge ethnic categorization schema, ethnicity is more rooted in shared ancestry, language, and culture and may be more appropriate for research about disparities in health [7]–[9].

Although there are a broad range of ethnic groups in the United States, the literature on potential differences between the health of different ethnic groups is limited almost exclusively to the health of Hispanics, the largest of these groups [10]. We focus here on the health of Arab Americans (AA), ethnic minorities who trace their ancestral, linguistic, or cultural heritage to one of 22 Arab countries [11]. Numbering around 3.5 million in the US during the 2001 census, evidence suggests that this population is growing [12]. As with many other ethnic groups, there are several reasons why a focus on the health of AAs in the US is warranted. Although the literature in the area is limited and there is little consensus about the population burden of high-impact diseases among AAs [11], extant studies suggest that AAs may have higher risk of cardiovascular disease, cancer incidence, and smoking than Whites [13]–[17]. Moreover, immigration [18], acculturation [19], and discrimination [20], all social exposures that may be disproportionately associated with the AA experience [20], may be important determinants of health and disease among AAs.

However, despite its growth and the substantial media focus on this population coincident with recent geopolitical events (including the terrorist attacks of September 11, 2001 and the subsequent wars in Afghanistan and Iraq) there has been limited attention paid to the health and well-being of AAs in the public health or medical literature [11]. To our knowledge, there are no studies that have calculated life expectancy among AAs, or considered population-based all-cause, cause-specific, and/or age-specific mortality in a complete (including children and adults) sample of AAs in any US state. In this study, we use data on all deaths in the state of Michigan, the state with the largest proportion of AAs in the US [21] between 1990–2007 to compare life expectancy, and all-cause, cause-specific, and age-specific mortality among AAs relative to non-ethnic (non-Hispanic and non-Arab) Whites.

## Methods

### Data

Data were collected about all deaths in the US state of Michigan from 1990 to 2007 from state death records collected by the Michigan Department of Community Health (MDCH). International Classification of Diseases (ICD) “underlying cause of death” codes were used to determine underlying causes of death. ICD-9 codes were used before 1998, and ICD-10 was used after 1998. Classifications used for this analysis included “septicemia”, “cancer- overall”, “diabetes”, “cardiac disease”, “cerebrovascular disease”, “pneumonia/flu”, “chronic respiratory disease”, “accident”, and “homicide”. We also obtained data on the race, ethnicity, age, and gender of all decedents. Only deaths among AAs and non-Arab and non-Hispanic Whites were considered to focus on disparities in mortality between this minority ethnic group relative to the majority ethnic group in the US. We determined White race by next-of-kin reported race from mortality data, and Arab ethnicity by Arab ancestry, which has been used in other studies about the health of AAs [22]–[25].

We used the American Factfinder Database [26] and the Integrated Public Use Microdata Series [27] to obtain Census 2000 population data. These population data were used as estimates of the population at approximately the midpoint of the time period of interest. In order to describe the underlying population of interest, namely Arabs and non-Arab and non-Hispanic Whites residing in the state of Michigan, we also collected information from the 2000 US Census on marital status (married, separated, divorced, widowed, and never married); education (none, less than 11th grade, general equivalency diploma (GED) or equivalent, and some college or greater); household annual income (negative, <$20,000, $20,001–$40,000, $40,001–$60,000, $60,001–$80,000, and greater than $80,000); gender (male vs. female); and age (10–19, 20–24, 25–44, 45–65, 65 and older) among Arabs and non-Arab and non-Hispanic Whites.

This study was reviewed by the Institutional Review Board of the Michigan Department of Community Health and the Health Sciences Institutional Review Board of the University of Michigan.

### Analysis

First, we calculated univariate statistics stratified by ethnicity and gender to describe the underlying population of interest. Second, we conducted bivariate chi-square tests, stratified by gender, to identify statistically significant (p<0.05) differences between Arabs and non-Arab and non-Hispanic Whites in the distribution of demographic characteristics. The Integrated Public Use Microdata Series18 interface was used to obtain population distributions of characteristics and to carry out bivariate chi-square tests [27].

Third, we calculated life expectancy stratified by ethnicity and gender using the life table method. Life tables allow us to calculate the probability of survival by age, and therefore the remaining life expectancy at each age from the sample. This method is commonly used when assessing differential mortality and/or life expectancy across demographic groups [28]. Fourth, we calculated age-adjusted all-cause mortality rates per 100,000, as well as age-specific mortality rates and age-adjusted cause-specific mortality rates by gender and ethnicity. Because our sample included all deaths in our study population, neither formal tests of significance nor confidence intervals were employed.

## Results

Table 1 shows univariate statistics and bivariate chi-square tests comparing demographic factors among AA and non-Arab and non-Hispanic White Michigan residents in 2000, stratified by gender. Among males, AAs were better educated (p<0.01), were more likely to be married (p<0.01), reported higher household incomes (p<0.01), and were older (p<0.01) than their non-Arab and non-Hispanic White counterparts. Among females, AAs reported higher household incomes (p<0.01) and were better educated (p<0.01) than non-Arab and non-Hispanic White females. AA females were also more likely to be 65 years or older than non-Arab and non-Hispanic White females.

**Table 1:** Demographic characteristics of Arab-American and non-Arab and non-Hispanic White males and females in Michigan, 2000.

	Males			Females		
	Arab ethnicity	White∧	X^2^p‵	Arab ethnicity	White∧	X^2^p‵
Total population	17,543	3,993,193		18,185	4,113,173	
Education			<0.01			<0.01
none	3.2	4.1		4.7	3.7	
<11 grade	28.0	31.2		28.7	29.0	
GED or equivalent	15.6	23.1		19.8	26.0	
Some college or greater	53.3	41.5		46.8	41.4	
Household income*			<0.01			<0.01
negative	0.3	0.5		1.1	0.6	
<20,000	8.5	9.4		13.0	13.5	
20–40,000	16.2	20.5		18.1	21.4	
40–60,000	19.8	22.1		18.9	20.8	
60–80,000	16.2	17.5		13.8	16.0	
>80,000	39.0	30.0		35.1	27.7	
Marital status			<0.01			0.12
married	51.5	44.7		40.9	43.4	
seperated	1.7	2.3		1.9	2.2	
divorced	5.0	7.1		8.6	8.7	
widowed	1.4	2.0		9.3	8.5	
single, never married	40.5	43.8		39.2	37.2	
Age			<0.01			0.05
0–4	4.4	6.7		6.6	6.2	
5–19	17.8	22.2		20.4	20.2	
20–24	8.0	7.7		5.7	7.1	
25–44	31.3	28.8		26.5	27.9	
45–64	23.7	23.5		21.8	23.2	
65+	14.7	11.1		19.0	15.4	

*in US dollars per year.

∧Non-Arab and non-Hispanic Whites.

‵Indicates p-values from X^2^ tests of the relation between each overall covariate (including all categories) and ethnicity.

Table 2 shows life expectancy at birth, average annual age-adjusted mortality rates per 10,000 overall and by major cause of death, as well as age-specific mortality by age group among AAs and non-Arab and non-Hispanic Whites in Michigan between 1990 and 2007. Life expectancy among both AA males and females was lower than that among non-Arab and non-Hispanic Whites. Among AA males, life expectancy at birth was 72.8 years, as compared to 74.8 years among non-Arab and non-Hispanic White men. Among AA females, life expectancy at birth was 78.7 as compared to 80.1 among non-Arab and non-Hispanic White females. All-cause mortality was higher among both AA males and females relative to non-Arab and non-Hispanic White males and females. Among males, age-specific mortality among AAs was higher at every age group except 25–44 years old than among non-Arab and non-Hispanic Whites. Among females, the age-specific mortality rate among AAs was higher than that among non-Arab and non-Hispanic Whites at both age extremes (<5 and ≥45 years old). With regard to cause-specific mortality, AA men had higher mortality than non-Arab and non-Hispanic White men due to septicemia, cancer, diabetes, cardiac disease, cerebrovascular disease, and homicide, and lower mortality than non-Arab and non-Hispanic White men due to chronic respiratory disease. AA women had higher mortality than non-Arab and non-Hispanic White women due to cancer, diabetes, cardiac disease, and cerebrovascular disease and lower mortality than non-Arab and non-Hispanic White women due to chronic respiratory disease.

**Table 2:** Life expectancy at birth and average annual all-cause, cause-specific, and age-specific mortality rate per 100,000 among Arab ethnicity and non-Arab and non-Hispanic Whites in Michigan, 1990–2007 by ethnicity and gender.

	Male		Female	
	Arab ethnicity	White∧	Arab ethnicity	White∧
Life expectancy at birth*	72.8	74.8	78.7	80.1
All-cause mortality	1509	1210	968	819
<5	188	172	175	135
5–19	44	39	20	22
20–25	135	116	24	43
25–44	131	175	76	88
45–64	789	746	496	465
65+	8306	6273	5383	4399
Septicemia	17	8	9	7
Cancer	299	246	192	166
Diabetes	52	27	32	21
Cardiac disease	465	344	284	219
Cerebrovascular disease	77	58	63	53
Pneumonia/Flu	19	17	10	10
Chronic respiratory disease	47	55	27	35
Accident	46	45	20	22
Homicide	18	3	2	2

*Life expectancy at birth calculated using the life table method.

∧ Non-Arab and non-Hispanic White.

## Discussion

In a study of all deaths in the state of Michigan among AAs and non-Arab and non-Hispanic Whites between 1990–2007, we found that both AA males and females had lower life expectancy than non-Arab and non-Hispanic Whites by 2 and 1.4 years, respectively, and that all-cause mortality was higher among AAs of both sexes than among non-Arab and non-Hispanic Whites. Mortality due to infectious diseases, cancer, diabetes, cardiac disease, cerebrovascular disease, and homicide were higher among AA relative to non-Arab and non-Hispanic White males, and mortality due to chronic respiratory disease was lower among AA males relative to non-Arab and non-Hispanic White males. AA females had higher mortality than non-Arab and non-Hispanic White females due to cancer, diabetes, cardiac disease, and cerebrovascular disease and lower mortality than non-Arab and non-Hispanic White females due to chronic respiratory disease. Age-specific mortality was higher among AAs than among non-Arab and non-Hispanic Whites among all age groups except males aged 25–44 years and females aged 5–44 years.

### Comparisons with the extant literature

Our findings contrast with a comparable study, which considered cause-specific and age-specific mortality among AA adults in Michigan between 1999–2001. Dallo and colleagues [29] found that although AA men (aged 25 or older) had higher all-cause mortality, and cause-specific mortality to heart disease, stroke, diabetes mellitus, pneumonia and influenza, and kidney disease relative to Whites, AA women (aged 25 and older) had lower all-cause mortality and cause-specific mortality for all causes considered (heart disease, cancer, stroke, chronic lower respiratory disease, diabetes mellitus, pneumonia and influenza, kidney disease, and chronic liver disease and cirrhosis) than Whites. This contrasts with our findings, which demonstrated that AA men and women generally had higher all-cause mortality, as well as higher chronic disease mortality than their White counterparts.

Our findings are more comparable to those of two previous studies in select samples of AAs in California, which have documented proportional mortality among AAs relative to US-born non-Hispanic Whites [30], [31]. Nasseri [30] found that proportional mortality ratios for coronary heart disease and diabetes were higher among first generation AAs relative to US-born non-Hispanic Whites in California, while those for chronic respiratory diseases were lower [30]. A similar study explored proportional mortality among first generation AAs relative to US-born non-Hispanic Whites in Orange County and Los Angeles County, California, and found similar observations about common causes of death among AAs relative to Whites [31].

Our work differs from these past studies and furthers the current understanding of the mortality experience of AAs in several ways. First, studies by Nasseri and colleagues rest on proportional mortality, and must be interpreted by considering mortality rates among AAs, as well as among Whites in a given context. Importantly, this relational characteristic of proportional mortality estimates limits comparisons of mortality among AAs with other groups in the same temporal and spatial context, or with Arab groups in different temporal or spatial contexts, limiting our ability to assess ecological differences or trends in AA mortality. Here we provide life expectancy and population-based all-cause, age-specific, and cause-specific mortality rates among AAs that are absolute, are not affected by mortality characteristics among Whites, and allow for cross-ethnic, spatial, and temporal comparisons.

While Dallo and colleagues estimated all-cause, age-specific, and age-adjusted cause-specific mortality among AA adults in their study [29], our work offers a substantial contribution above and beyond the extant work in several important ways. First, whereas Dallo and colleagues consider only data about adults, we considered mortality among children, as well. For this reason, our all-cause mortality estimates account for childhood mortality (as true measures of all-cause mortality should). Given that our findings suggest that AA children may have higher mortality than their White counterparts, the inclusion of data about children in our analysis and exclusion of this data from the previous analysis by Dallo and colleagues may explain differences between our all-cause mortality metrics and those of Dallo and colleagues relative to Whites. Moreover, the inclusion of data about children in our analysis allowed us to estimate life expectancy metrics among AAs, which, to our knowledge, have not been considered in other studies. Second, Dallo and colleagues used an Arab name algorithm to attribute Arab ethnicity in their analysis [29], whereas we used self-reported Arab ancestry. As several studies have demonstrated, Arab name algorithms are only moderately sensitive and/or specific in detecting Arab ancestry [30], [32]. The use of this method to attribute Arab ethnicity in the previous analysis by Dallo and colleagues is an important limitation. For example, Dallo and colleagues found substantial differences in mortality between AA men and women in their study relative to Whites among. This is likely explained by their use of an Arab name algorithm to attribute Arab ethnicity—as women who marry may change their surnames after marriage, intermarriage of Arab women to non-Arab men as well as of non-Arab women to Arab men may decrease both the sensitivity and specificity of the algorithm among men and women. This suggests that the findings from Dallo and colleagues among women may be inaccurate, explaining why they found such a substantial gender dimorphism in the mortality experience of AAs relative to Whites [29]. Rather, in our analysis, we employed self-reported Arab ancestry, which does not suffer from the same methodological limitations as the name algorithm.

### Differences in life expectancy and all-cause mortality between Arab Americans and non-Arab and non-Hispanic Whites

The observation that AA men and women had lower life expectancy by about 2 years, and higher all-cause mortality rates compared to non-Arab and non-Hispanic Whites was unexpected given higher education and income among AAs relative to non-Arab and non-Hispanic Whites, and well-established evidence that individuals of higher socioeconomic status in the US generally have lower mortality [33]–[35]. These findings are also surprising when contrasted with findings regarding mortality among other ethnic minority groups in the US, such as Hispanic-Americans [36], [37] and Asian-Americans [38] that have demonstrated advantages in life expectancy and all-cause mortality relative to Whites.

There are two possible explanations for our observations of higher mortality rates among AAs compared to other ethnic and non-Arab and non-Hispanic White groups. First, features particular to Arab American cultural traditions may explain some of the differences in mortality between AA and Whites. We found that chronic disease mortality was disproportionately high among AAs relative to Whites. This may be explained, in part, by worse dietary and physical activity habits among this group relative to Whites. For example, in a study of the relation between acculturation and diabetes risk among AAs in Michigan, Jaber and colleagues [39] showed that acculturation was associated with lower risk for diabetes among AAs. The authors reasoned that AA cultural adherence may be associated with lower physical activity and a less healthy diet. This is supported by another study [40], which demonstrated a relatively high prevalence of cardiovascular disease risk factors among a largely foreign-born sample of Arab Americans in southeast Michigan, including in particular, low physical activity levels and poorer diets with higher fat intake. It is plausible that Arab cultural traditions, specifically the paucity of a culture of explicit exercise, as well as a more corpulent image of beauty, may promote chronic disease risk factors, and ultimately mortality among this group relative to Whites [40], [41].

Second, experiences of discrimination, as well as acculturative stress among AAs may explain some of this observation. Several studies have demonstrated that experiences of discrimination and acculturative stress may influence the physical and mental health of ethnic minority groups [18], [20], [42]–[47], including Arab Americans [18], [20], [45]–[47]. Smith and colleagues suggest that discrimination can influence ethnic minority health in three fundamental ways: It can directly harm the wellbeing of ethnic minority individuals. Moreover, it can systematically worsen their socioeconomic status, and therefore, their health. And finally, it can exacerbate their recognition of relative disadvantage, and thereby negatively impact their health [48]. By contrast, acculturative stress has been defined as the mental or emotional strain of the process of change that individuals or populations may undergo when they come in contact with a different culture [49], [50], and has been shown to compromise ethnic minority health [49], [51]–[53]. A recent immigrant group receiving considerable attention in the public discourse, it is possible that discrimination against AAs, along with pressure to acculturate out of fear of discrimination, may be particularly strong among this group. Therefore, it is plausible that disparities in life expectancy and all-cause mortality between AAs and Whites may be explained, in part, by experiences of discrimination and acculturative stress among AAs.

We found that AA men and women had lower mortality to chronic respiratory disease than Whites. Although the limited literature about smoking behavior among AAs relative to Whites has not yet reached consensus, the most recent study about smoking behavior among AAs relative to Whites found that AAs may be less likely to smoke than their White counterparts [54]. It is plausible, therefore, that differences in smoking behavior between AAs and Whites may explain this finding.

### Differences in age-specific and cause-specific mortality between Arab Americans and non-Arab and non-Hispanic Whites

We found that among males, AAs had higher age-specific death rates compared to non-Arab and non-Hispanic Whites at all ages except 25–44 years. Among females, AAs had higher age-specific death rates than non-Arab and non-Hispanic Whites at both age extremes: under 5 years and older than 45 years. The finding that under 5 mortality was higher among AAs than among non-Arab and non-Hispanic Whites is surprising considering a small literature suggesting that AAs have better perinatal health markers than Whites in the US, exhibiting lower rates of preterm birth, low birth weight, and very low birth weight, in particular [19], [22]–[25]. To our knowledge, there have been no studies concerning the health of AA children following the early neonatal period. Our observation that AAs had higher death rates above 65 years was also not expected, considering that elderly Hispanic and Asian-Americans—comparable ethnic minority groups to AAs—have been shown to have mortality advantages compared to Whites [36]–[38].

These findings may be explained by the dynamics of immigrant health. Several studies in the global health literature have documented a “double burden” of disease in low and middle income countries, whereby populations in these contexts suffer from a high burden of infectious diseases, as well as a growing burden of chronic diseases [55]–[57]. Mortality among populations suffering from high burdens of infectious disease is characterized by high child (under 5) and adolescent mortality, as these diseases disproportionately kill young people with poor nutrition and underdeveloped immune systems [58], [59]. Alternatively, mortality among populations suffering from high burdens of chronic diseases is characterized by higher mortality among the elderly [60].

Comparing mortality by cause between different global regions stratified by age group and gender, Murray and Lopez [61] found that both the probability of death to infectious and tropical diseases among males and females 1–15 years old, as well as the probability of death to chronic diseases among males and females aged 60–70 was higher in the Middle Eastern Crescent as compared to countries with established market economies, like the US. AAs, recent immigrants to the US, may show mortality patterns more similar to those in Middle Eastern countries from whence they recently arrived than those in the US. It is plausible, therefore, that our findings may be explained by a higher infectious disease mortality burden among AA children, as well as a higher chronic disease mortality burden among AA elderly compared to Whites of similar age in the US. Although the frequency of deaths among AAs were too few to support stratification by age and cause of death, the high mortality burden to both infectious diseases and chronic diseases among AAs relative to non-Arab and non-Hispanic Whites, as well as the age-specific patterning of mortality among AAs relative to non-Arab and non-Hispanic Whites, support this explanation.

### Limitations

There are several limitations to consider when interpreting our findings. First, our findings may not generalize to other contexts within the US, as our data come from only one US state. However, Michigan is the only state, to our knowledge, which collects data about Arab ancestry in vital registry files, and therefore presents an uncommon opportunity to analyze population-based mortality among AAs. Second, while our estimates of all-cause and cause-specific mortality were stratified by ethnicity and gender and adjusted for age, we did not account for any other factors that could have confounded the relation between ethnicity and all-cause, cause-specific, and/or age-specific mortality. Third, we are limited by the accuracy of death certificate data collected from vital registry files as well as by population statistics from Census 2000 data, although it has been shown that discrepancies in vital statistics and census data may offset one another [62]. Fourth, given the relatively small proportion of deaths to AAs in vital registry data, it was necessary to average death registry data to yield stable results. Therefore, our mortality data were derived from 18 years of vital registry death certificate whereas our population denominator estimate was from just one year. This may have biased age-specific mortality rates reported here, as population age structure may change with time.

### Implications

Our observations suggest important disparities in the mortality experiences of AAs relative to non-Arab and non-Hispanic Whites. Despite better education and higher incomes, our findings that, on average, AAs can expect to live about 2 years shorter than their non-Arab and non-Hispanic White counterparts, and that AAs are more likely to die of high burden chronic diseases as well as infectious diseases are causes for concern. Given these findings, public health departments in areas with significant numbers of AA minorities might consider interventions that target chronic disease risk factors among AAs, as well curb the spread of infectious diseases among this ethnic group. Moreover, clinicians of AA patients should counsel their patients about modifiable risk factors for diabetes and cardiovascular disease among adults, as well as strategies for mitigating the spread of infectious agents among children. These findings can also be used to educate AAs about particular health concerns within their community.

The findings also have several important implications for future research. First, it is plausible that other rapidly growing ethnic groups also have different health indicators, including mortality, than non-ethnic whites, and inquiry into the health of these groups seems warranted. Second, because little is known about the prevalence of diabetes, cardiovascular, cerebrovascular, and infectious diseases among AAs, prospective studies of AA health are crucial. Such studies might clarify the roles of social exposures, such as immigration and discrimination, as well as acculturation, in the production of health and disease among this ethnic group. Third, investigators interested in the health of AAs in the US may also consider the mortality experience of AA populations in other contexts in the US. Fourth, particular attention should be paid to AAs at both extremes of age, under 5 and older than 45, so as to understand the causes and consequences of health and disease among these age-groups, shown to be particularly disadvantaged relative to Whites.
